# Influence of Single Experience with Intraoperative Near-Infrared Autofluorescence on Postoperative Parathyroid Insufficiency after Thyroidectomy - A Preliminary Clinical Study

**DOI:** 10.7150/ijms.72886

**Published:** 2022-07-18

**Authors:** Paweł Domosławski, Marcin Adamiecki, Łukasz Antkowiak, Krzysztof Paśko, Mariusz Chabowski, Jędrzej Grzegrzółka, Michał Zrąbkowski, Jacek Białecki, Ryszard Antkowiak

**Affiliations:** 1 Department of General, Minimally Invasive and Endocrine Surgery, Wroclaw Medical University, Wrocław, Poland.; 2 Department of Orthopedic, Oncological and Reconstructive Surgery, Medical University of Silesia, Regional Hospital, Sosnowiec, Poland.; 3 Department of Pediatric Neurosurgery, Medical University of Silesia, Katowice, Poland.; 4 Department of General and Endoscopic Surgery, EMC Euromedicare Hospital Wroclaw, Wrocław, Poland.; 5 Department of Surgery, 4th Military Teaching Hospital, 50-981 Wroclaw, Poland.; 6 Division of Anesthesiologic and Surgical Nursing, Department of Nursing and Obstetrics, Wroclaw Medical University, Wroclaw, Poland.; 7 Department of Histology and Embryology, Wroclaw Medical University, Wroclaw, Poland.; 8 Department of General, Minimally Invasive and Trauma Surgery, Franciszek Raszeja Memorial Hospital, Poznan, Poland.; 9 Department of General and Vascular Surgery, 3rd Provincial Hospital, Rybnik, Poland.

**Keywords:** near-infrared autofluorescence, thyroidectomy, high-volume surgeon, fluobeam

## Abstract

**Introduction:** Total thyroidectomy has become the most common thyroid procedure. This treatment method results in most postoperative hypocalcemia (PH) and hypoparathyroidism (HPT) cases due to the unwitting removal of the parathyroid glands (PTGs). Near-infrared autofluorescence (NIRAF) is a new method that helps identify PTGs. This study aimed to determine whether short-term experience with intraoperative NIRAF may influence postoperative complications after thyroidectomy.

**Materials and methods:** Overall, 65 patients who underwent thyroidectomy by one high-volume surgeon were enrolled in the study between March 2018 and August 2021. In August 2020, the surgeon performed four operations using the NIRAF device. After that experience, the technique of operating and preserving PTGs has been totally changed. Postoperative serum calcium (Ca) and parathormone (PTH) concentrations were measured. Using retrospective study analysis, we assessed the rate of PH and HPT.

**Results:** There was no statistically significant difference in Ca (p = 0.1612) and PTH (p = 0.3590) concentrations between groups operated on before and after the NIRAF experience. The serum concentrations of Ca and PTH of all patients were positively correlated (r = 0.4074; p = 0.0022) as well as the Ca concentration and age of patients (r = 0.3292; p = 0.0116), respectively.

**Conclusions:** These findings suggest that short-term NIRAF experience, and changing attitude to preserving PTGs does not affect thyroidectomy outcomes, even when utilized by a highly experienced high-volume thyroid surgeon. However, continuous use of NIRAF might enhance treatment outcomes, particularly for surgeons with limited experience.

## Introduction

Total thyroidectomy (TT) is the most common thyroid procedure, not only in cases of neoplastic changes but also in most cases of nodular goiter and particularly in Graves' disease. This procedure is statistically responsible for postoperative hypocalcemia (PH), which is transient in 13-36% of patients or remains permanent in 1-4% of patients 1-7. Patients with prolonged hypoparathyroidism (HPT) treated longer than six months are more susceptible to kidney insufficiency and, for unknown reasons, have a higher risk of developing neoplasms and cardiac diseases, and they live shorter. This complication is directly connected with surgery due to vascular damage, inadvertent resection, or autotransplantation of unwittingly removed parathyroid glands (PTGs), even by high-volume thyroid surgeons 8.

PTGs are very difficult to detect because of their size and similarity to surrounding fat tissue and localization. Thus, even very experienced surgeons have difficulty detecting them during thyroidectomy 9. Eye-based detection is the current standard for PTG recognition. There are many approaches to creating a method that improves the detection of the PTGs. An intravenous injection of methylene blue was used in the past, but this technique did not make the PTGs so obvious and was not accepted by surgeons 10. Frozen tissue section analysis and intraoperative parathormone (PTH) evaluation have also been considered. However, they are not commonly used because they are invasive, generate costs, and depend on professional expertise 11.

Intraoperative indocyanine green angiography (ICG) is a promising method for identifying PTGs. ICG is not specific to PTG parenchyma, although its use can help establish the vascularity of PTGs. However, this visualization may be dependent on bleeding. Above all, there is no recommendation concerning the dosage and timing of ICG injections. Consequently, further studies must be conducted to determine the value of this method 11,12. By contrast, a new technique that uses fluorescence to detect PTGs intraoperatively without contrast agents has been developed, which may be more widely used in surgical departments.

Fluorescence occurs when a substance is illuminated by light of higher energy, which subsequently emits light of lower energy that can be detected on the monitor using a special camera built-in one system 8,9. PTGs are excited by near-infrared (NIR) light waves at a length of 785 nm, reemitting at a wavelength between 820-830 nm with about 11 times more intensity than surrounding tissues. This wave can be detected and visualized on a screen by near-infrared spectroscopy or an NIR camera 8,13,14. This phenomenon is based on intrinsic fluorophores in some tissues that possess native fluorescence or autofluorescence.

The NIR autofluorescence (NIRAF) of the PTGs is much stronger than that of surrounding tissues [14] and is based on the presence of intrinsic fluorophores in PTGs. The real fluorophore remains unknown, but autofluorescence depends on the cellularity of PTGs, the expression of calcium-sensing receptors (CaSR), or the presence of pseudo-colloid 15. The NIRAF device shows the localization of but does not provide information about the viability of PTGs. Intravenous injection of ICG can provide such information 11,12. In November 2018, the American Food and Drug Administration (FDA) approved the marketing of two devices to detect PTGs, the Fluobeam 800 Clinic Imaging Device (image-based) and the Parathyroid Detection PTeye (probe-based) System 9.

This study aimed to retrospectively estimate the improvement in postoperative outcomes through a single experience with intraoperative NIRAF by a high-volume thyroid surgeon.

## Material and methods

### Study settings

Following institutional review board approval, patients who underwent thyroidectomy at the Department of General, Minimally Invasive and Endocrine Surgery at the Wroclaw Medical University and Euromedicare Hospital in Wroclaw (Poland) between March 2018 and August 2021 were included in the study. Individuals were divided into two groups: those operated before NIRAF imaging experience (Group 1) and those operated after NIRAF imaging (Group 2). All of the operations were performed by one surgeon classified as a high-volume thyroid surgeon who performs more than 40 cases of thyroid operations per year 16. In August 2020, the surgeon performed four operations using the NIRAF device. After this single experience he totally changed the way of localizing and preserving PTGs. The next operations he started to perform firstly by opening the sides of the thyroid gland and then localizing and preserving of PTGs. Previous operations he performed by starting the thyroidectomy first and then finding PTGs during operation. The aim of the study was to determine whether a single experience with an NIRAF device could influence his subsequent operations on patients from Group 2.

### Study model

Using retrospective study analysis, we assessed the rate of PH and HPT using international guidelines. Measurements of calcium (Ca) serum and PTH were performed on the first postoperative day. All patients with serum Ca levels <2.2 mmol/l (normal range 2.2-2.75 mmol/l) were considered to have hypocalcemia. All patients with PTH levels <15 pg/ml (normal range 15-65 pg/ml) were considered to have HPT and received oral Ca carbonate with vitamin D analogs. The length of hospital stay was also noted.

### Surgical procedure and AF monitoring

**Figure 1:**
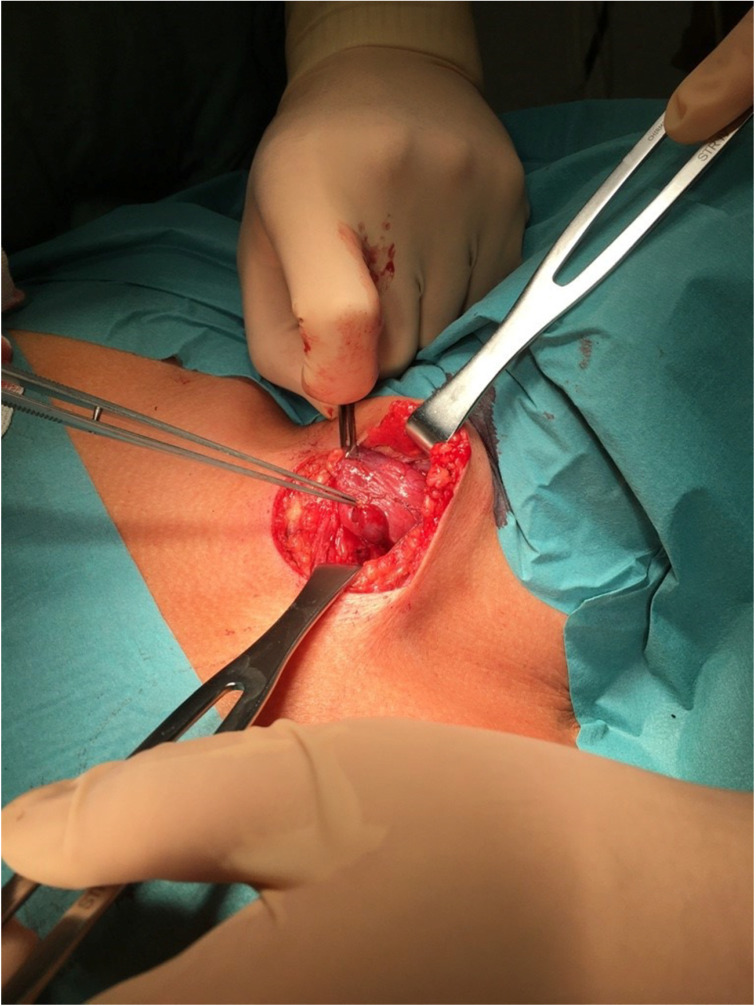
Intraoperative view of incision site with the visible thyroid gland and pointed tool toward PTG. To compare with Figure 2, which shows the same thyroid and PTG on a Fluobeam monitor in gray scale.

All procedures were performed under general anesthesia. From the transverse cervicotomy, the surgeon separated the neck muscles and visualized the thyroid gland **(Figure 1)**. Thyroidectomy was performed after visualization or detection by neuromonitoring both recurrent laryngeal nerves and PTGs.

**Figure 2:**
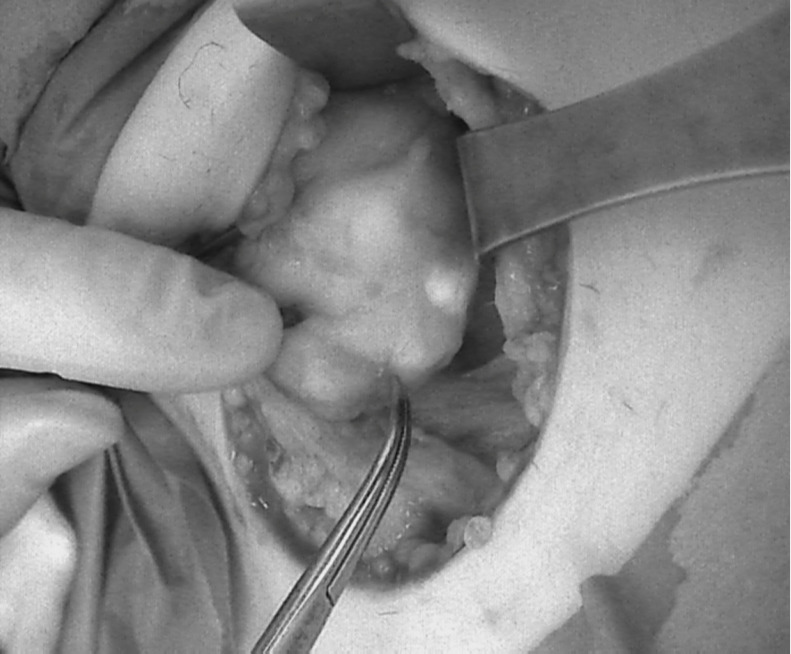
The picture seen on the Fluobeam monitor: intraoperative view of marked PTG as a brighter spot with surrounding darker tissue, compared with Figure 1, which shows the same picture of the thyroid and PTGs.

In four cases, image-based NIRAF was used to confirm the identification of the PTGs. These operations were performed in August 2020. Those patients were excluded from the comparison. His next operations were performed with much more careful technique, preserving much more surrounding PTGs tissue. A Fluobeam LX (Fluoptics, Grenoble, France) device was equipped with a laser camera, console, and display monitor, and it was calibrated before each usage. The measurements were performed approximately 20 cm from the operating field, with lights partially switched off in the operating room. The surgeon was also equipped with a magnifying system. The identified PTGs were visualized on the screen as bright tissue, in contrast to the surrounding darker tissue **(Figure 2)**. The procedure prolonged the operation time by no more than a few minutes. Serum Ca and PTH levels were measured on the first postoperative day.

### Statistical analysis

All statistical analyses were performed using Prism 5.0 software (GraphPad, La Jolla, CA, USA). The Kolmogorov-Smirnov test was used to evaluate the normality assumption of the examined groups. The Fisher's exact test was used to compare a difference in the proportion of cases in each category. The unpaired t-test or Mann-Whitney test was used to compare the differences of age and examined serum concentrations of examined markers in all groups. Spearman's rank correlation coefficient was used to analyze the existing correlations. The results were considered statistically significant when p <0.05.

## Results

Overall, 65 patients were included in this study. A total of 37 patients from Group 1 were compared with 28 patients from Group 2. Forty-nine patients (75.38%) were considered to have hypocalcemia, and 16 patients (24,62%) were considered to have HPT, both with a higher percentage in Group 2 (85.71% for Ca and 21.42% for PTH). Most patients in each group were female, and they were of similar ages. The main reason for the operation in both groups was thyroid carcinoma. There were a similar number of total thyroidectomies (TOTs) with local lymphadenectomies in both groups (45,95% vs 46,43%), these groups were of statistical significance (p = 0.0206). In total, 17 patients from Group 1 and 13 patients from Group 2 were classified using the Bethesda diagnostic system for thyroid cancer. The studied groups were homogenous regarding chosen patients' characteristics, such as age, sex, diagnosis, type of treatment, and Bethesda diagnostic category (p >0.05). Detailed characteristics of the study groups are presented in Table 1.

**Figure 3:**
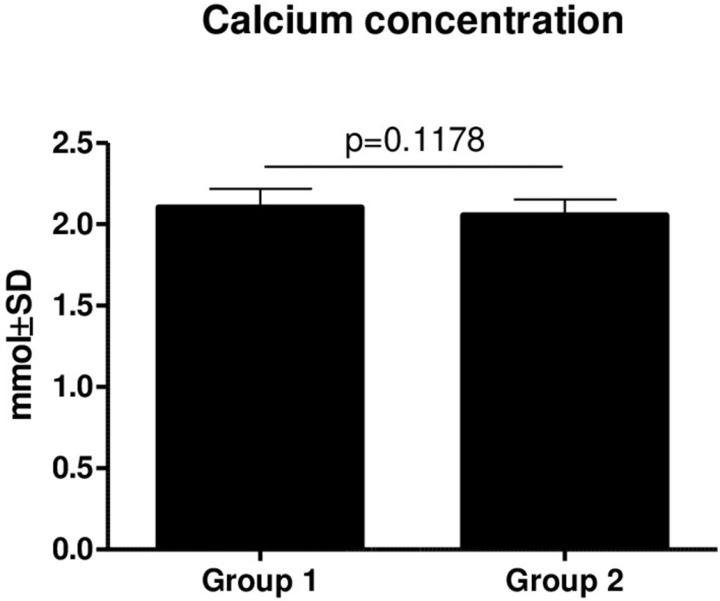
Comparison of Ca concentrations in Group 1 and Group two (mean ± SD).

**Figure 4:**
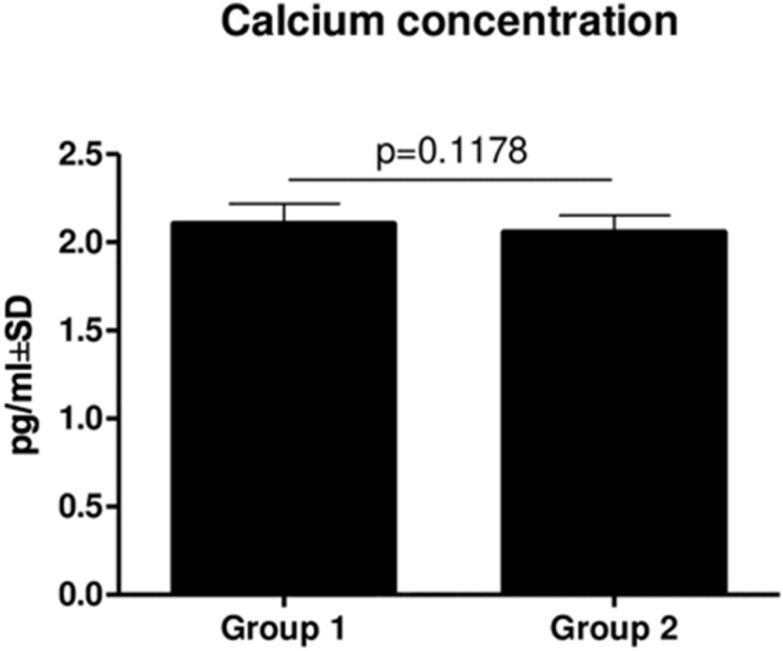
Comparison of PTH concentrations in Group 1 and Group 2 (mean ± SD).

There was no statistically significant difference in Ca (p = 0.1178) **(Figure 3)** and PTH (p = 0.3590) **(Figure 4)** concentrations between Groups 1 and 2. However, there was a slightly decreasing tendency of the PTH concentration in patients from Group 2, but it was not statistically significant.

**Figure 5:**
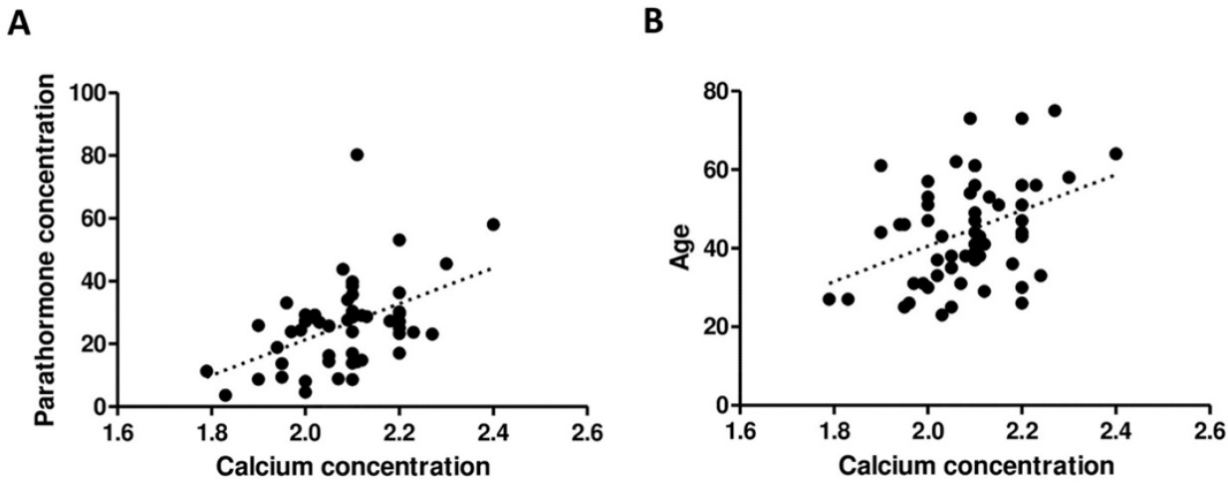
Correlation between PTH and Ca concentration (A) as well as between age and Ca concentration (B).

The serum concentrations of Ca and PTH of all patients were positively correlated (r = 0.4074; p = 0.0022) as well as the Ca concentration and age of patients (r = 0.3292; p = 0.0116), respectively **(Figure 5)**.

## Discussion

NIR light sources can penetrate tissue as deep as 5 mm, which makes NIR light sources optimal for fluorescence imaging. By contrast, fluorescence caused by short wavelengths (e.g., ultraviolet light) can only be detected within a few microns on the surface of the tissue 9. The NIRAF of PTGs is much stronger than that of surrounding tissues and has a peak emission at a wavelength of approximately 820 nm 17. The real fluorophore remains unknown, but autofluorescence depends on the cellularity of PTGs, the expression of CaSR, or the presence of pseudo-colloid 15.

Two different approaches to NIRAF have been gaining greater acceptance: probe-based and image-based techniques. In probe-based techniques, such as FDA-cleared PTeye, surgeons use a sterile probe for tissue measurement with the NIR laser. The console gives surgeons audio-visual feedback to determine the PTG location 10,18,19. An image-based NIRAF technique based on the camera visualizes the operation field on a display monitor that highlights a tissue of interest 9.

Even though this method requires a constant correlation between monitor and surgical field images, we found that method to be easy and intuitive 11. Since the development of NIRAF, many studies have shown its effectiveness. Image-based PTG detection ranges from 94% to 100% 17. Falco et al. reported that using NIR camera imaging helps detect more PTGs during surgery. About 3.7 PTGs were detected with the use of an NIR camera in contrast to the average 2.5 glands recognized by the naked eye (p <0.0001) 15,20. Similar results were found by Falco et al. 15,20 who showed an increased number of PTGs identified from 2.6 in classical methods to 3.5 using an NIR camera. Studies have shown that in 33% to 46% of cases, PTGs can be recognized by image-based NIRAF before being seen by the naked eye 21,22. The more PTGs are recognized, the better the postoperative outcomes 23. Benmiloud et al. 23 showed that using NIR imaging was associated with an increased mean number of recognized PTGs, a lower rate of PTG autotransplantation, and a significantly lower rate of PH (5.2% vs. 20.9%).

In this study, we present the effect of a short-term NIRAF experience by a high-volume thyroid surgeon on the rate of PH and HPT. Thus, we assessed how the short-term experience with actual localization of PTGs visualized by Fluobeam changing of operating technique which result in future operations performed by the same surgeon in finding and preserving PTGs.

We conclude that there was no statistically significant difference in the rate of the mentioned complications in Group 1 versus Group 2. We also found no difference in postoperative complication rates between groups operated on with and without NIRAF imaging. At present, most studies have focused on the sensitivity, accuracy, and specificity of NIRAF imaging in PTG identification, especially in comparison to probe-based techniques 10,24. The literature lacks data focusing on the educational aspects of NIR imaging.

This topic is crucial, particularly as NIRAF imaging is still developing and not many hospitals have such devices. A gold standard for PTG recognition is still based on visual inspection of the thyroidectomy specimen. A short-term experience with NIRAF techniques could probably have an influence on surgeon's skills to better understand a real localization of parathyroid glands, to achieve better postoperative results.

Our study demonstrates that short-time NIRAF experience does not affect treatment outcomes even when utilized by highly experienced high-volume thyroid surgeons. However, NIRAF may be a more helpful device for low-experienced surgeons without extensive knowledge of the topography of PTGs. Accordingly, some authors have pointed out NIRAF as a potentially beneficial training device that could lead to better understating of the PTG intraoperative anatomy 10,25. Solórzano et al. 9 reported that surgeons who were present during the operation but did not use the NIRAF for PTG detection showed concurrent improvement of their skills, reducing iatrogenic hypocalcemia. Therefore, although the literature suggests the potential clinical utility of NIRAF, we hypothesize that more prolonged NIRAF usage in thyroidectomies is necessary to evaluate the actual influence of NIRAF on postoperative PTG functioning. This is because even high-volume surgeons have a problem with PTG detection, and only continuous use of the NIRAF technique can improve their operation results.

### Study limitations

The major limitation of our study is its retrospective nature, lack of randomization, and small sample size of patients operated on with the use of the NIRAF device. This study also lacks data about the number of recognized PTGs with histological confirmation of the tissue and preoperative serum concentrations of Ca and PTH. Group 2 included two patients with preoperatively diagnosed hyperthyroidism, reaching borderline statistical significance compared to Group 1. Nevertheless, the study was designed as preliminary research that will be extended to a larger number of thyroid centers in Poland, concentrating on PTG preservation after thyroidectomy.

## Conclusions

Our study proves that short-term NIRAF experience does not affect treatment outcomes, even when utilized by highly experienced, high-volume thyroid surgeons. However, continuous use of NIRAF might enhance treatment outcomes, particularly for surgeons with limited experience. Hence, we suggest that surgical wards should consider acquiring permanent NIRAF units for improving experience and data as well as for better postoperative PTG outcomes after thyroidectomy.

## Figures and Tables

**Table 1:** Participants' characteristics in both study groups.

Characteristic	Group 1 (N=37)	Group 2 (N=28)	Total (N=65)	p-value
Age *Mean (range)*	46 (25-75)	43 (21-73)	45 (21-75)	0.1939
Sex *n (%)*				
Females	34 (91.89)	26 (92.86)	60 (92.31)	1.0000^#^
Males	3 (8.11)	2 (7.14)	5 (7.69)	
Diagnosis *n (%)*				
Thyroid cancer	17 (45.95)	13 (46.43)	30 (46.15)	0.0206^#^
Other non-toxic goiters	20 (54.05)	10 (35.71)	30 (46.15)	
Hyperthyroidism	0	5 (17.86)	5 (7.70)	
Type of treatment *n (%)*				
Tot	20 (54.05)	15 (53.57)	35 (53.85)	0.9692^#^
Tot+lymphadenectomy	17 (45.95)	13 46.43)	30 (46.15)	
Bethesda diagnostic category *n (%)*				
V	15 (88.24)	9 (69.23)	24 (80)	0.1972^#^
VI	2 (11.76)	4 (30.77)	6 (20)	

*Note:* p - Fisher exact test.

## Abbreviations

Ca: calcium
CaSR: calcium-sensing receptors
FDA: American Food and Drug Administration
HPT: hypoparathyroidism
ICG: indocyanine green angiography
NIRAF: near-infrared autofluorescence
PH: postoperative hypocalcemia
PTH: parathormone
TT: total thyroidectomy
